# Phase angle, impedance ratio, and athletic performance of young adult Japanese track and field throwers

**DOI:** 10.14814/phy2.70835

**Published:** 2026-04-02

**Authors:** Akihisa Hikita, Kazushige Oshita, Ryota Myotsuzono, Satoki Murai

**Affiliations:** ^1^ Department of Sport Science Kyushu Kyoritsu University Kitakyushu Fukuoka Japan; ^2^ Department of Human Information Engineering Okayama Prefectural University Soja Japan

**Keywords:** BIA, bioelectrical impedance analysis, body composition, fat‐free mass, muscle quality

## Abstract

This study investigated the relationship between World Athletics (WA) points and body size, body composition, phase angle (PhA), and impedance ratio (IR) among young adult Japanese university track and field throwing athletes. Twenty‐five female and 39 male athletes (11 and 14 javelin throwers, 5 and 13 discus throwers, 6 and 7 hammer throwers, 3 and 5 shot putters; and WA points: 852.5 ± 90.3 and 831.8 ± 105.4, respectively) underwent body composition, PhA, and IR measurements using bioelectrical impedance analysis, and their best competition records for the season were examined. No significant relationship was found between WA points and body size. While no significant relationship was found between WA points and fat‐free mass in females, a weak relationship was observed in males. Therefore, the importance of muscle mass in throwing performance may differ across the four disciplines. However, PhA and IR were significantly and substantially related to WA points in both sexes. Furthermore, the higher WA points group had significantly higher PhA and lower IR values than the lower WA points group, with large effect sizes. These results suggest that PhA and IR, which are indicators of muscle quality, may be useful markers of throwing performance.

## 1 INTRODUCTION

Track and field throwing events include shot put, discus, javelin, and hammer throw. Performance in these events requires well‐developed biomechanical skills, that is, throwing techniques, and high biological potential, that is, body size (the most widely used anthropometric measurements are body mass and height; Gibson, 2005), body composition, and fitness. Athletes in all four throwing disciplines rely on ballistic power and maximal extremity strength to enhance their performance (Zhao et al., 2023). Therefore, many throwers' training programs are dedicated to enhancing their muscle strength, power, and fast force production (Judge, 2007; Stone et al., 2003; Zaras et al., 2016; Zhao et al., 2023). Accordingly, resistance training is regularly performed by throwers to increase their muscle strength and power, leading to significant increases in fat‐free mass (FFM), especially in muscle groups directly involved in specific throwing events (Zaras et al., 2021). Consequently, university track and field throwers have a higher FFM than their counterparts in other track and field events (Hirsch et al., 2016).

Changes in FFM in track and field throwers correlated significantly with changes in rate of force development and maximum strength, suggesting that FFM contributes to rapid force development (Zaras et al., 2021). However, some studies indicated that muscle mass is not correlated with throwing performance. Aikawa et al. (2020) investigated the relationship between World Athletics (WA) points (mean, 810 ± 122) and body mass or body composition in a mixed group of four disciplines of Japanese university throwers. The study showed no significant relationship between WA points and body mass or body composition. Therefore, while a certain muscle mass may be necessary to achieve high throwing performance, the relationship between body mass or muscle mass and throwing performance may not be consistent in well‐trained athletes. A recent review of the biological determinants of track and field throwing performance discussed FFM and performance (Zaras et al., 2021). Discus and javelin implements are lighter than shot put and hammer throw implements, meaning that discus and javelin throws are more dependent on movement velocity and rate of force development than on maximum strength, based on the force–velocity relationship (Zaras et al., 2021). Since achieving higher performance in shot put and hammer throw requires greater muscle force output due to increased muscle mass, FFM appears to be closely associated with performance in these events. However, while reaching a certain level of FFM is necessary for higher performance in discus and javelin throwing, it may be of lesser importance in well‐trained athletes (Zaras et al., 2021).

Popular methods of measuring and evaluating body composition are skinfold measurements (ISAK methodology) and dual‐energy X‐ray absorptiometry (DXA) (Mathisen et al., 2023). Furthermore, bioelectrical impedance analysis (BIA) is also one of the popular methods. BIA analysis requires adherence to certain ground rules, including the use of equations specific to the target population and the absence of fluid imbalances, body shape abnormalities, and extremely large or small body sizes (Gonzalez et al., 2019). However, BIA is also a relatively low‐cost, easy‐to‐use, and portable method (Ellis, 2000). This technique estimates the body composition based on the impedance values of a weak alternating current (AC) passing through the body. BIA can also enable the measurement of phase angle (PhA) and impedance ratio (IR), and the ESPEN (European Society for Clinical Nutrition and Metabolism) “Blue” Book introduces them as follows (Gonzalez et al., 2019). The PhA is currently considered a global marker of tissue health. PhA is calculated from the resistance (*R*) and reactance (Xc) components of the impedance (*Z*), and it is a reasonably good indicator of body cell mass (BCM), but it also indicates cell membrane integrity and function. IR is another indicator using a multifrequency BIA device. Resistance at low‐frequency AC (<50 kHz) is evaluated to determine extracellular water (ECW), while resistance at high‐frequency AC (>100 kHz), where cell membranes are penetrated, is evaluated to determine total body water (TBW). IR is calculated as the ratio of high‐frequency to low‐frequency AC impedance. Under normal conditions, the ratio of impedance at 200 to 5 kHz is always less than 1; however, when the cell membrane function is altered or impaired, this ratio approaches unity. Therefore, these are considered indicators of BCM, soft‐tissue cellular integrity (Di Vincenzo et al., 2019; Lukaski et al., 2017), ECW/intracellular water (ICW) ratio (Di Vincenzo et al., 2019), and cellular health (Kworweinski et al., 2025). Previous study reported that although PhA is lower and IR is higher in older adults than in younger adults, age‐related differences in PhA and IR are greater than those in muscle mass (Oshita, Hikita, Myotsuzono, & Ishihara, 2025). Therefore, muscle mass may not necessarily reflect cellular health.

A recent meta‐analysis of athletes from various sports revealed a significant positive relationship between PhA and lower limb muscle power (counter‐movement jump) (Cirillo et al., 2023). Another review article suggests that, since PhA is an objective indicator of cell membrane damage, tissue injury, and healing responses, it can be used to assess the healing process and return to physical activity in athletes (Nescolarde et al., 2023). Furthermore, changes in muscle strength and jump height in national‐level athletes have been reported to be significantly related to changes in ICW (Silva et al., 2014). Therefore, PhA or IR in sports may be a promising approach for assessing “muscle quality” in athletes (Di Vincenzo et al., 2019). While some reports suggested that these indicators showed no significant differences across sports disciplines (Di Vincenzo et al., 2019), others indicated that athletes specializing in velocity/power have a higher PhA than endurance athletes (although the magnitude of this difference is small) (Campa et al., 2022). Furthermore, muscle strengthening results in greater increases in PhA than endurance training (Marra et al., 2021). Therefore, athletes competing at a higher level in sports requiring explosive power, such as track and field throwing events, may also have a higher PhA. Although there are limited reports on IR and sports performance, IR has been found to be lower in athletes (dancers) than in control groups, and even lower at the professional level than at the amateur level (Ballarin et al., 2021). Consequently, IR is also considered an index related to sporting performance. However, the relationship among throwing performance, PhA, and IR remains unclear. While body composition measurements using the BIA method “estimate” body composition by applying variables such as age, gender, height, and others to a prediction equation, PhA and IR utilize the “raw” parameters obtained directly from BIA. Therefore, if a significant relationship exists between WA points and PhA or IR in competitive throwers, these could serve as BIA indicators independently of predictive equations related to throwing performance. Therefore, this study investigated the relationships among WA points, body size, body composition, PhA, and IR in Japanese university track and field throwing athletes. Hereafter, we have used the term “throwing” or “thrower” to refer to the combined “throwing” of the four track and field disciplines.

## 2 METHODS

### 2.1 Participants

The participants were 64 throwing athletes from universities and graduate schools in Japan. Of them, 25 were female (19 ± 1 years; 11 javelin throwers, 5 discus throwers, 6 hammer throwers, and 3 shot putters), and 39 were male (19 ± 1 years; 14 javelin throwers, 13 discus throwers, 7 hammer throwers, and 5 shot putters). Prior to the study, the participants were informed of the content and provided written consent for body composition measurements and competition records. Body size and body composition were measured in November 2024, and the best competition results achieved during that season were investigated. This study was reviewed and approved by the Research Ethics Committee of Kyushu Kyoritsu University (approval number: 2022‐08) and conducted in accordance with the ethical principles of the Declaration of Helsinki.

McKay et al. proposed a tier‐based classification system to standardize terminology in sports science literature due to the lack of well‐defined classification criteria, such as elite or trained status, within exercise science and physiology (McKay et al., 2022). Their classifications are as follows: Tier 0: Sedentary; Tier 1: Recreationally Active; Tier 2: Trained/Developmental; Tier 3: Highly Trained/National Level; Tier 4: Elite/International Level; and Tier 5: World Class. Based on this classification, most of the participants in this study fall into Tier 2 to Tier 3, with some included in Tier 4.

### 2.2 Body size and body composition

Body mass, fat mass (FM), FFM, TBW, PhA, and IR were measured using a standing 8‐electrode multifrequency BIA device (MC‐780A‐NS; TANITA, Japan). The device measures *R* and Xc of the whole body, upper limbs, and lower limbs by applying AC of less than 90 μA at frequencies of 5, 50, and 250 kHz from electrodes placed on plantar feet and palms. As this BIA device measures FFM with high correlation to DXA measurements in both males and females, regardless of their level of physical activity (*r* = 0.976; concordance coefficient: 0.955), it is considered an effective tool for measuring body composition among young adults (Verney et al., 2015). Body height was recorded to the nearest 0.1 cm using a stadiometer, while body mass index (BMI) was calculated by dividing body mass in kilograms by height in meters squared.

Participants wiped the palms of their hands and soles of their feet with alcohol‐free wet wipes to clean and moisten the electrode contact areas. They then stepped onto the electrode portion of the machine and grasped the electrodes with both palms to enable the measurements. Although this BIA measurement involves gripping the electrodes, throwing athletes may have calluses on their palms due to the nature of their sport. However, plantar calluses in overweight adults have been reported not to affect BIA measurements (Roekenes et al., 2015). Participants were asked to ensure that their arms, trunk, and inner thighs were not touching while holding their arms straight down. They were asked to confirm that they had not consumed excessive food or fluids, were not dehydrated, and had not engaged in strenuous exercise prior to the measurements; they were asked to ensure that at least 3 h had passed since their last meal, and to urinate and defecate beforehand.

PhA was calculated using Xc and *R* at 50 kHz AC as the arctangent of (Xc/*R*) × (180°/*π*), and evaluated as an absolute value. *Z* was calculated from *R* and Xc, whereas IR was calculated from the *Z* of 5 and 250 kHz AC. PhA and IR were evaluated as whole body values by averaging the measurements from the right and left sides, as arm values by averaging the measurements from both arms, and as leg values by taking measurements from both legs. Furthermore, the impedance index (Ht^2^/*Z*) was calculated by dividing the whole body impedance (Ω) at each AC frequency by the square of the height (cm). As this study examined both single‐armed and double‐armed throwing disciplines, averaging values for the right and left sides was adopted for all raw BIA variables.

### 2.3 Competition records

WA Scoring Tables are a set of benchmark tools that convert athletic performances (such as times, distances and heights) into a standardized point system. This allows for fair comparisons to be made between different events and genders. Each participant's best performance in each track and field event between April and November in 2024 was assessed. These records were then converted to WA points by referring to the Scoring Tables for Athletics (2022 REVISED EDITION) (World Athletics, 2022). The WA points for the participants in this study were as follows: javelin throwers were 733–1083, discus throwers were 663–985, hammer throwers were 745–925, and shot putters were 694–757 for males; and javelin throwers were 677–889, discus throwers were 692–859, hammer throwers were 811–1042, and shot putters were 851–888 for females.

### 2.4 Data analysis

The measurements were analyzed separately for the males and females. Each measurement value is presented as the mean ± standard deviation (SD) for all participants, along with the maximum, median, and minimum values.

The relationships between the WA points and each measurement variable were examined using regression analysis. The regression coefficient, referencing the table summarized by Overholser and Sowinski (2008), is classified as follows: *R* ≤0.19, negligible relationship; *R* = 0.20–0.39, weak relationship; *R* = 0.40–0.69, substantial relationship; and *R* ≥0.70, strong relationship.

Participants were divided into higher and lower WA point groups based on the median value (804 for males and 851 for females). As the number of participants in this study was odd for both males and females, the group sizes were unequal. According to McKay's classifications of athletes' competitive levels, WA points at Tier 3 are 932 ± 138 for males and 977 ± 91 for females (McKay et al., 2022). Therefore, the mean minus 1 SD for Tier 3 is 794 for males, which is lower than the median in this study, and 886 for females, which is higher. Thus, participants at the median point were assigned to the higher points group for males and the lower points group for females. Consequently, most participants in the lower points group were in Tier 2 and most in the higher points group were in Tier 3, including some in Tier 4. Since this study divided participants at the median competitive level, it was predicted that the measurement values in each group would not follow a normal distribution. Therefore, the Mann–Whitney *U‐*test was used to compare the FFM, TBW, PhA, IR, and Ht^2^/*Z* between two groups, and the effect size was calculated using Cohen's *d* value.

The statistical analyses were performed using the StatFlex software (ver. 7.0.10; Artec, Osaka, Japan). The statistical significance level was defined as *p* < 0.05, and a significant trend was defined as 0.05 ≤ *p* < 0.10.

## 3 RESULTS

Table 1 shows the mean ± SD, maximum, median, and minimum values for each measurement variable.

**TABLE 1:** Mean ± standard deviation, maximum, median, and minimum values for each measurement variable.

	Male		Female	
	Mean ± SD	(max, med, min)	Mean ± SD	(max, med, min)
WA point	832.9 ± 105.7	(1083, 804, 663)	852.8 ± 90.3	(1042, 851, 677)
Height (cm)	177.2 ± 5.4	(188.3, 177.2, 166.0)	166.7 ± 5.1	(176.9, 166.1, 155.5)
Body mass (kg)	90.7 ± 14.0	(116.6, 88.6, 70.2)	72.3 ± 8.5	(91.9, 71.0, 57.2)
BMI (kg/m^2^)	28.9 ± 4.2	(36.2, 28.3, 21.4)	26.0 ± 3.1	(32.2, 25.3, 21.8)
%BF (%)	19.3 ± 5.2	(29.1, 19.4, 10.4)	31.4 ± 5.0	(41.8, 30.1, 24.4)
FM (kg)	18.1 ± 7.3	(31.0, 17.3, 7.9)	23.1 ± 6.4	(38.4, 21.4, 14.6)
TBW (kg)	47.4 ± 5.3	(61.1, 46.3, 39.8)	34.8 ± 2.9	(40.3, 34.6, 28.8)
FFM (kg)				
Whole body	72.5 ± 7.4	(86.4, 70.9, 60.8)	49.2 ± 3.2	(53.9, 49.7, 42.6)
Upper limb	7.0 ± 1.2	(9.6, 7.0, 4.6)	4.6 ± 0.9	(6.8, 4.6, 1.9)
Lower limb	27.7 ± 2.8	(34.0, 27.1, 24.3)	18.8 ± 1.5	(21.5, 18.8, 16.1)
PhA (deg.)				
Whole body	7.35 ± 0.43	(7.99, 7.34, 6.40)	6.47 ± 0.37	(7.37, 6.41, 5.90)
Upper limb	7.19 ± 0.45	(8.05, 7.24, 6.30)	6.43 ± 0.40	(7.36, 6.38, 5.68)
Lower limb	7.53 ± 0.58	(8.86, 7.53, 6.22)	6.71 ± 0.44	(7.49, 6.70, 5.86)
IR				
Whole body	0.742 ± 0.015	(0.774, 0.740, 0.719)	0.772 ± 0.013	(0.790, 0.773, 0.743)
Upper limb	0.746 ± 0.015	(0.780, 0.745, 0.716)	0.772 ± 0.013	(0.795, 0.775, 0.744)
Lower limb	0.736 ± 0.020	(0.784, 0.736, 0.697)	0.764 ± 0.016	(0.769, 0.765, 0732)
Ht^2^/*Z* (cm^2^/Ω)				
5 kHz	53.0 ± 6.3	(70.3, 52.3, 43.0)	38.9 ± 3.5	(46.3, 39.5, 30.9)
50 kHz	62.5 ± 7.7	(83.2, 61.3, 51.1)	44.7 ± 4.1	(52.3, 45.4, 35.3)
250 kHz	71.5 ± 8.8	(94.8, 69.9, 58.3)	50.5 ± 4.7	(58.8, 50.8, 39.9)

Abbreviations: BF, body fat; BMI, body mass index; FFM, fat‐free mass; FM, fat mass; Ht, body height; IR, impedance ratio; PhA, phase angle; SD, standard deviation; TBW; total body water; WA, World Athletics; *Z*, impedance.

**FIGURE 1:**
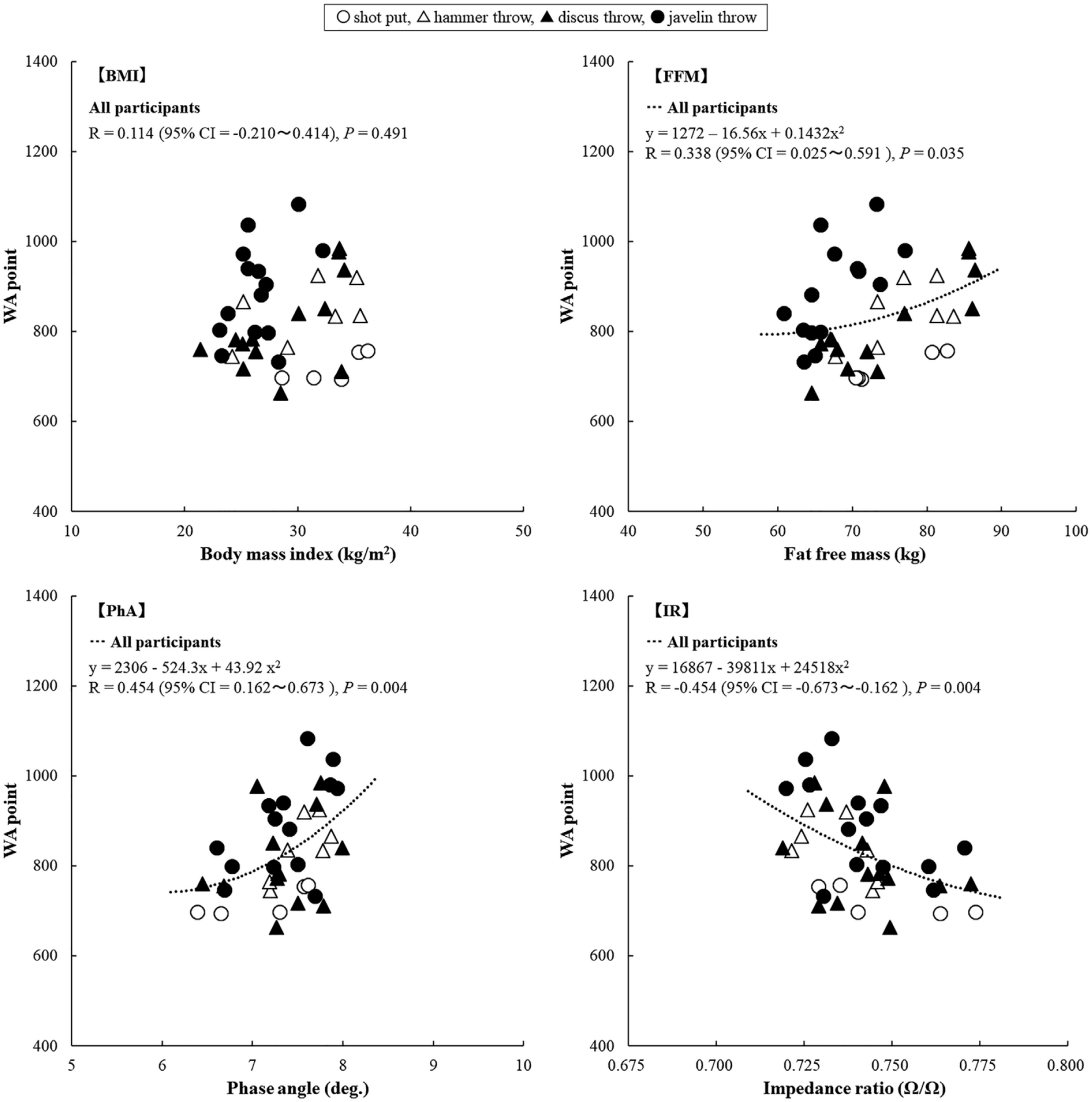
Relationship between WA points and BMI, FFM, PhA, and IR in male participants. BMI, body mass index; CI, confidence interval; FFM, fat‐free mass; IR, impedance rate; PhA, phase angle; WA, World Athletics.

The WA points in males exhibited no significant relationship with body size or FM. Figure 1 shows the relationship between the WA points for males and BMI, FFM, PhA, and IR (whole body). The WA points exhibited no significant relationship with BMI. WA points exhibited a significant relationship with FFM (*R* = 0.34, *p* = 0.04), PhA (*R* = 0.45, *p* < 0.01), and IR (*R* = −0.45, *p* < 0.01). However, the relationship with FFM was weak, whereas those with PhA and IR were substantial (Figure 1). These results indicate that, although male WA points are not significantly related to body size, they are weakly related to FFM and substantially related to PhA and IR. Although there was no significant relationship between WA points and lower limb FFM (*R* = 0.22, *p* = 0.18), there was a tendency toward significance in relation to upper limb FFM (*R* = 0.30, *p* = 0.08). Although WA points exhibited no significant relationship with lower limb PhA and IR (*r* = 0.27, *p* = 0.10 and *r* = −0.27, *p* = 0.10), it exhibited a significant and substantial relationship with upper limb PhA and IR (*r* = 0.47, *p* < 0.01 and *r* = −0.44, *p* < 0.01).

**FIGURE 2:**
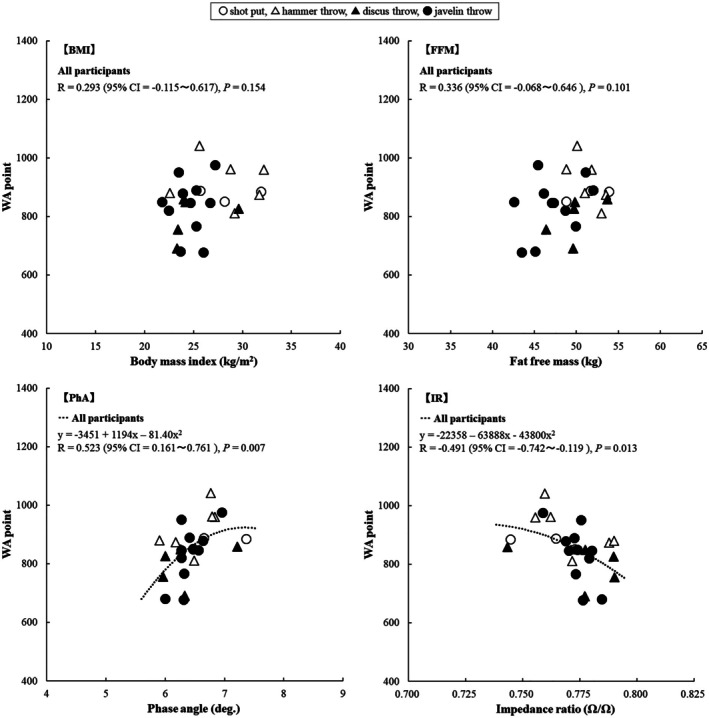
Relationship between WA points and BMI, FFM, PhA, and IR in female participants. BMI, body mass index; CI, confidence interval; FFM, fat‐free mass; IR, impedance rate; PhA, phase angle; WA, World Athletics.

The WA points for females exhibited no significant relationships with body size or FM. Figure 2 shows the relationship between the WA points for females and BMI, FFM, PhA, and IR. The WA points exhibited no significant relationship with BMI or FFM. However, WA points presented a significant relationship with PhA (*R* = 0.52, *p* < 0.01) and IR (*R* = −0.49, *p* = 0.01). These results indicate that, while female WA points are not significantly related to body size or body composition, they are significantly and substantially related to PhA and IR. Further, although WA points exhibited no significant relationship with upper and lower limb FFM (*r* = 0.25, *p* = 0.24, and *r* = 0.28, *p* = 0.17), it exhibited a significant and substantial relationship with upper limb and lower limb PhA (*r* = 0.50, *p* = 0.01, and *r* = 0.41, *p* = 0.04) and IR (*r* = −0.52, *p* < 0.01 and *r* = −0.41, *p* = 0.04).

Table 2 compares FFM, TBW, PhA, IR, and Ht^2^/*Z* by WA points in males. The mean WA points value was 743.8 ± 38.1 in the lower points group (*n* = 19) and 917.5 ± 74.3 in the higher points group (*n* = 20). FFM, PhA, and IR exhibited no significant differences between groups in the lower limbs (PhA and IR exhibited a significant trend). However, in the whole body and upper limbs, FFM and PhA were significantly higher and IR was significantly lower in the higher points group compared to the lower points group. The effect sizes for these variables were approximately *d* = 0.5 or 0.8 for FFM, while *d* ≥0.9 for PhA and IR. These results indicate that although FFM, PhA, and IR in whole body and upper limb differ significantly according to WA point, effect sizes were larger in PhA and IR. Furthermore, although there was no significant difference in Ht^2^/*Z* at 5 kHz between the groups, there was a significantly higher tendency for Ht^2^/*Z* at 50 kHz and 250 kHz in the higher points group.

**TABLE 2:** FFM, PhA, IR and impedance of the higher versus lower WA point groups in males.

	Lower points		Higher points		*U*‐test (*p*)	Effect size (*d*)
	Mean ± SD	(max, med, min)	Mean ± SD	(max, med, min)		
WA point	743.8 ± 38.1	(799, 755, 663)	917.5 ± 74.3	(1083, 916, 804)		
TBW (kg)	45.2 ± 3.3	(51.8, 45.3, 40.3)	49.4 ± 6.0	(61.1, 49.4, 39.8)	0.02	0.84
FFM						
Whole body	69.7 ± 5.2	(82.7, 68.0, 63.5)	75.3 ± 8.2	(86.4, 75.3, 60.8)	0.02	0.81
Upper limb	6.6 ± 1.1	(8.3, 6.8, 4.6)	7.4 ± 1.2	(9.6, 7.5, 5.5)	0.04	0.76
Lower limb	27.0 ± 2.0	(31.3, 26.8, 24.3)	28.5 ± 3.4	(34.0, 28.6, 24.4)	0.25	0.54
PhA (deg.)						
Whole body	7.15 ± 0.42	(7.79, 7.26, 6.40)	7.53 ± 0.35	(7.99, 7.59, 6.61)	<0.01	0.99
Upper limb	6.96 ± 0.39	(7.41, 7.02, 6.30)	7.40 ± 0.40	(8.05, 7.46, 6.34)	<0.01	1.12
Lower limb	7.37 ± 0.57	(8.29, 7.25, 6.34)	7.68 ± 0.57	(8.86, 7.77, 6.22)	0.09	0.56
IR						
Whole body	0.748 ± 0.014	(0.774, 0.746, 0.729)	0.735 ± 0.012	(0.771, 0.735, 0.719)	<0.01	1.01
Upper limb	0.754 ± 0.013	(0.773, 0.750, 0.736)	0.739 ± 0.015	(0.780, 0.737, 0.716)	<0.01	1.02
Lower limb	0.741 ± 0.020	(0.778, 0.742, 0.704)	0.730 ± 0.019	(0.784, 0.728, 0.697)	0.09	0.56
Ht^2^/*Z* (cm^2^/Ω)						
5 kHz	51.1 ± 4.1	(57.1, 51.5, 44.1)	54.7 ± 7.5	(70.3, 53.4, 43.0)	0.14	0.59
50 kHz	59.9 ± 4.93	(68.3, 60.5, 52.1)	65.0 ± 9.0	(83.2, 64.2, 51.1)	0.07	0.69
250 kHz	68.3 ± 5.7	(77.9, 69.0, 59.4)	74.5 ± 10.3	(94.8, 73.9, 58.3)	0.05	0.73

Abbreviations: FFM, fat‐free mass; Ht, body height; IR, impedance ratio; PhA, phase angle; SD, standard deviation; TBW, total body water; WA, World Athletics; *Z*, impedance.

Table 3 compares FFM, TBW, PhA, IR, and Ht^2^/*Z* by WA points in females. The mean WA points value was 790.4 ± 68.2 in the lower points group (*n* = 13) and 920.4 ± 56.0 in the higher points group (*n* = 12). Whole body FFM and PhA were significantly higher and IR was significantly lower in the higher points group compared to the lower points group with large‐sized effects (*d* ≥1.0). Although upper and lower limb FFM exhibited no significant differences between groups (lower limb exhibited a significant trend), PhA and IR were significantly higher and IR was significantly lower in the higher points group compared to the lower points group with large‐sized effects (*d* ≥1.0). These results indicate that although upper and lower limb FFM were not significantly different according to the WA point, PhA and IR differ significantly with large‐sized effects. Furthermore, although there was no significant difference in Ht^2^/*Z* at 5 and 50 kHz between the groups, there was a significantly higher tendency in the higher points group for Ht^2^/*Z* at 250 kHz.

**TABLE 3:** FFM, PhA, IR and impedance of the higher versus lower WA point groups in females.

	Lower points		Higher points		*U*‐test (*p*)	Effect size (*d*)
	Mean ± SD	(max, med, min)	Mean ± SD	(max, med, min)		
WA point	790.2 ± 68.3	(851, 821, 677)	920.1 ± 56.0	(1042, 888, 859)		
TBW (kg)	33.7 ± 2.7	(38.0, 33.3, 28.8)	36.1 ± 2.7	(40.3, 35.6, 31.9)	0.04	0.91
FFM						
Whole body	47.8 ± 2.9	(53.0, 48.7, 42.6)	50.6 ± 2.8	(53.9, 51.1, 45.4)	0.01	1.04
Upper limb	4.7 ± 1.0	(5.7, 4.8, 2.0)	5.3 ± 1.0	(7.3, 5.4, 3.4)	0.23	0.54
Lower limb	19.5 ± 1.6	(22.6, 19.6, 17.2)	20.6 ± 1.5	(22.9, 20.4, 18.0)	0.09	0.69
PhA (deg.)						
Whole body	6.29 ± 0.19	(6.56, 6.31, 5.96)	6.67 ± 0.42	(7.37, 6.66, 5.90)	0.01	1.17
Upper limb	6.26 ± 0.27	(6.54, 6.34, 5.68)	6.61 ± 0.45	(7.36, 6.56, 6.04)	0.03	0.96
Lower limb	6.51 ± 0.31	(7.09, 6.55, 5.86)	6.92 ± 0.46	(7.49, 7.02, 5.93)	0.02	1.05
IR						
Whole body	0.778 ± 0.006	(0.790, 0.778, 0.770)	0.765 ± 0.015	(0.790, 0.765, 0.743)	0.01	1.16
Upper limb	0.778 ± 0.009	(0.795, 0.777, 0.767)	0.765 ± 0.014	(0.788, 0.763, 0.744)	0.01	1.12
Lower limb	0.772 ± 0.011	(0.796, 0.772, 0.755)	0.757 ± 0.016	(0.791, 0.756, 0.732)	0.01	1.09
Ht^2^/*Z* (cm^2^/Ω)						
5 kHz	38.0 ± 3.8	(44.7, 39.2, 30.9)	39.9 ± 3.0	(46.3, 40.1,35.0)	0.22	0.56
50 kHz	43.4 ± 4.4	(50.4, 44.6, 35.3)	46.1 ± 3.5	(52.3, 45.9, 40.2)	0.12	0.68
250 kHz	48.9 ± 4.9	(56.6, 50.3, 39.9)	52.2 ± 3.9	(58.8, 51.8, 45.6)	0.09	0.75

Abbreviations: FFM, fat‐free mass; Ht, body height; IR, impedance ratio; PhA, phase angle; SD, standard deviation; TBW, total body water; WA, World Athletics; *Z*, impedance.

## 4 DISCUSSION

For this BIA measurement device, the manufacturer Tanita's Instruction Manual specifies an accuracy of 2% for impedance measurement (Tanita Europe B.V, n.d.). This study observed significant trend differences in Ht^2^/*Z* between groups at 50 and 250 kHz for males, and at 250 kHz for females, with the following values (lower vs. higher points group): 59.9 ± 4.93 versus 65.0 ± 9.0, 68.3 ± 5.7 versus 74.5 ± 10.3, and 48.9 ± 4.9 versus 52.2 ± 3.9, respectively. The technical variability for impedance of this device is less than the observed difference between groups in this study (>2%). Furthermore, a previous study has found that the hydration indices (sum of ICW and ECW) measured by this device exhibited between‐day reliability of 36.58 and 36.64 kg, and within‐day reliability of 36.58 and 36.72 kg, with differences in both cases being less than 1% (Mohammed‐Qadah et al., 2024). The between‐group differences in TBW (lower vs. higher points group) in this study were 45.2 ± 3.3 versus 49.4 ± 6.0 kg for males and 33.7 ± 2.7 versus 36.1 ± 2.7 kg for females. The within‐ or between‐day variability for body water content of this device is less than the observed difference between groups in this study (>1%). Therefore, the significant or significant trend differences observed between groups for each variable in this study are not considered to be due to measurement error.

This study found no significant relationship between FFM and WA point in females. Although a significant positive relationship was found between FFM and WA point in males, it was weak. Further, WA points also exhibited no significant relationship with upper and lower limb FFM (*p* ≥ 0.05). The relationship between body composition and throwing performance varies across disciplines (Zaras et al., 2021). Therefore, the relationship between FFM and throwing performance should be examined individually for each discipline. Furthermore, a recent review suggested that height is not the most decisive factor in performance among throwers at a similar level, although being tall enough is necessary from a biomechanical perspective (Zaras et al., 2021). Moreover, heavier athletes tended to perform better in the shot put and hammer throw events than lighter athletes, although the link between these two factors was weak (Zaras et al., 2021). Some reports indicated no significant relationship between body mass and performance in the shot put and hammer throw events (Kyriazis et al., 2009; Terzis et al., 2010). Therefore, FFM appears to have no or a weak relationship with throwing performance among athletes at certain competitive levels.

In contrast to FFM, whole body PhA and IR exhibited significant and substantial relationships with WA points. Further, a significant relationship was observed between WA points and PhA and IR only in the upper limbs for males, whereas a significant relationship was observed in both the upper and lower limbs for females, although the R value was greater for the upper limbs. Although it is thought that such differences by body part are due to the “throwing” motion involving the upper limbs in this discipline, the mechanisms underlying the differences in body composition and PhA/IR remain unclear. Many throwers' training programs focus on enhancing muscle strength, power and fast force production (Judge, 2007; Stone et al., 2003; Zaras et al., 2016; Zhao et al., 2023). As mentioned in the introduction, PhA is believed to reflect the integrity of the cell membrane. A higher PhA (i.e., fine cell membrane integrity) is considered beneficial for transmitting electrically evoked action potentials, and PhA has been reported to be related to voluntary and evoked muscle contractile properties (Hirata et al., 2023). Therefore, the higher points group had a high PhA, which enabled them to contract their muscles more strongly and quickly. This may have contributed to their higher competitive performance. Furthermore, TBW is calculated using *Z* at high‐frequency AC, while ECW is calculated using *Z* at low‐frequency AC (Gonzalez et al., 2019). The IR calculated using these impedances was significantly lower in the higher points group. This study also found no significant difference in Ht^2^/*Z* at 5 kHz between groups, whereas Ht^2^/*Z* at 250 kHz was higher in the higher points group. Therefore, while ECW demonstrated no group differences, TBW was higher in the higher points group, suggesting a larger intracellular component and a relatively smaller extracellular component. Furthermore, these ratios, IR, exhibited significant and substantial relationships with WA points. Although force generated in myofibres has to be transmitted laterally through the extracellular matrix to adjacent fibers (known as lateral force transmission in skeletal muscles) (Zhang & Gao, 2012), increasing extracellular matrix thickness decreases such force transmission (Zhang & Gao, 2014). Although this previous study focused on age‐related changes in muscle (Zhang & Gao, 2014), this study suggests that the smaller extracellular space in the higher points group may lead to faster force transmission and greater force generation. Consequently, participants with higher levels of competition may have smaller extracellular components, enabling them to contract their muscles more strongly and quickly.

Another possible reason why FFM and WA were unrelated was the potential for errors in athletes' body composition measurements obtained using the BIA method. Body composition is estimated by inserting values for age, height, body mass, gender, and other variables, as well as recorded resistances (*R*, Xc and *Z*), into a prediction equation. These equations have been developed for the general population and active individuals. However, although specific equations are available for athletes, they do not account for differences between individuals (Malá et al., 2018). Therefore, as body size varies by discipline, it is difficult to evaluate the body composition of different athletes using a single estimation formula. Indeed, previous studies have noted that BIA measurements are subject to random error due to differences in body size, geometry, and composition, resulting in non‐uniform conductivity (Lukaski & Raymond‐Pope, 2021; Moon, 2013). Consequently, the generalized equation is considered to overestimate and/or underestimate FFM and FM, depending on the athlete (Moon, 2013). However, PhA and IR utilize raw BIA measurements without employing estimation equations. The finding in this study that PhA and IR exhibited significant relationships with the WA point suggests that they may serve as BIA indicators relevant to throwing performance.

While this study found that PhA and IR were significantly related to WA points, the R‐values were not particularly high, at around 0.5. Therefore, it is important to note that an excellent PhA or IR does not necessarily guarantee high performance, as biomechanical factors (e.g., throwing techniques and body mechanics) also play a role. Throwing success requires well‐developed movement techniques (Bartlett, 1992; Bartlett & Best, 1988; Castaldi et al., 2022; Morriss & Bartlett, 1996; Schofield et al., 2022). Throwing athletes generally use the kinetic chain to transfer energy from their lower extremities through their pelvis, trunk, shoulders, arms, wrists, and hands into their implement, thereby generating maximum force (Meron & Saint‐Phard, 2017) and achieving high performance. Furthermore, the “run‐up” technique on a predetermined runway is a crucial factor in javelin throwing performance (Takigawa & Tauchi, 2023; Tauchi et al., 2012). Consequently, some participants had PhA and IR values similar to those in the higher points group despite having lower WA points (Figures 1 and 2). As this study did not measure indicators of muscle strength or power, future research should evaluate performance metrics independent of throwing technique (e.g., maximum strength or power tests, such as grip strength, vertical jump, and isokinetic force). However, a study of 185 Japanese males and females aged 18 found an *r* value of between 0.54 and 0.57 for the relationship between PhA (whole body and upper limbs) and grip strength (Oshita et al., in press). Furthermore, systematic reviews of various populations reveal that, while many studies confirm a significant relationship between PhA and muscle strength, the correlation coefficients range from *r* = 0.10 to 0.596 (or *r* = 0.153 to 0.425 after adjusting for factors such as weight, gender, and age) (Custódio Martins et al., 2022), or from *r* = 0.177 to 0.696 (Kuschel et al., 2022). Similarly, the relationship between IR (whole body and upper limbs) and grip strength in 18‐year‐old Japanese individuals was *r* = −0.56 to −0.53 (Oshita et al., in press). These values are comparable in strength to those observed with WA points in this study. Therefore, while PhA and IR may be significantly related to muscle strength, muscle power and athletic performance, the strength of this relationship may be weak to moderate.

In addition to the limitations mentioned in the discussion, this study has other limitations. First, although the BIA measurements were obtained in November (end of competition season), WA points were calculated using competition records from April to November. Therefore, there may have been a maximum gap of approximately 6 months between the athletes' best performance and the BIA data. Although PhA has been reported to be lower in the preseason than in the mid‐season or before competitions in several sporting events (Campa et al., 2020; Reis et al., 2020), no significant difference has been observed between mid‐ and end‐season PhA (Mascherini et al., 2015). Therefore, while the PhA measured at the end of the season in this study may not differ significantly from that measured at the mid‐season, the most preferable approach would be to measure BIA data alongside competition records. However, because some elite athletes dislike undergoing various measurements, particularly during competition, it is often difficult to perform BIA during every competition. Furthermore, explosive athletes repeatedly engage in high‐intensity exercise throughout the season. PhA remains significantly lower in the exercised limb than in the unexercised limb, even 168 h after high‐intensity eccentric exercise (Yamaguchi et al., 2024). Therefore, BIA data collected during the mid‐season may be relatively unstable due to variations in training exercises. Second, this study focused on Japanese athletes; however, PhA differs across racial groups (Barbosa‐Silva et al., 2005; Mattiello et al., 2020). Therefore, it is unclear whether the current findings apply to non‐Japanese or ‐Asian individuals. Third, although this study had a limited sample size, future studies will require a larger sample size across each discipline, as well as discipline‐specific analyses. Although this study examined four disciplines together, as outlined in the Methods section, it did not investigate the dominant and non‐dominant limbs. Therefore, if discipline‐specific analyses were performed, it would be possible to make comparisons between the dominant and non‐dominant limbs. Furthermore, a previous review article reported that the changes in PhA resulting from resistance training and detraining are greater than the changes in FFM (Lukaski & Raymond‐Pope, 2021). A previous study reported that individuals with higher levels of physical activity or athletic performance exhibit higher PhA, even when there are no differences in BMI or FFM (Oshita, Hikita, Myotsuzono, & Murai, 2025). This study also found that the effect sizes for PhA and IR were larger than those for FFM among groups at different competitive levels. While the participants of this study were primarily Tier 2 to Tier 3 athletes, expanding the sample size could facilitate the development of benchmark PhA and IR values for each tier. Finally, as this was a cross‐sectional study, future longitudinal studies examining changes in performance, PhA, and IR are necessary.

## 5 CONCLUSION

This study investigated the relationship between WA points and body size, body composition, PhA, and IR among Japanese university track and field throwers. No significant relationship was found between FFM and WA points in females, whereas a weak relationship was found in males. However, a significant relationship was found between WA points, PhA, and IR in both males and females. These results suggest that although FFM has no substantial relationship with WA points owing to the differing importance of the four throwing disciplines, indicators of muscle quality, such as PhA and IR, may be useful markers of throwing performance.

## AUTHOR CONTRIBUTIONS

**Akihisa Hikita:** Conceptualization; funding acquisition; investigation; methodology; project administration; resources; supervision; validation. **Kazushige Oshita:** Conceptualization; data curation; formal analysis; funding acquisition; methodology; project administration; supervision; validation; visualization. **Ryota Myotsuzono:** Funding acquisition; investigation; resources; validation. **Satoki Murai:** Data curation; formal analysis; funding acquisition; validation; visualization.

## FUNDING INFORMATION

This work was supported by the Okayama Foundation for Science and Technology and JSPS KAKENHI (grant number JP24K09636).

## CONFLICT OF INTEREST STATEMENT

The authors declare that they have no competing interests.

## ETHICS STATEMENT

This study was reviewed and approved by the Research Ethics Committee of Kyushu Kyoritsu University (approval number: 2022‐08), and informed consent was obtained from all the participants prior to the examination.

## Data Availability

The datasets generated and analyzed in the current study are available from the corresponding author upon reasonable request.
